# Supplementary material for: The Impact of Increased CO2 and Drought Stress on the Secondary Metabolites of Cauliflower (Brassica oleracea var. botrytis) and Cabbage (Brassica oleracea var. capitata)
Source: Plants (Basel). 2023 Aug 29;12(17):3098. doi: 10.3390/plants12173098 (PMC10490549; doi:10.3390/plants12173098)
Supplement: Supplementary file 1 [file plants-12-03098-s001.zip › plants-2546976-supplementary.pdf]

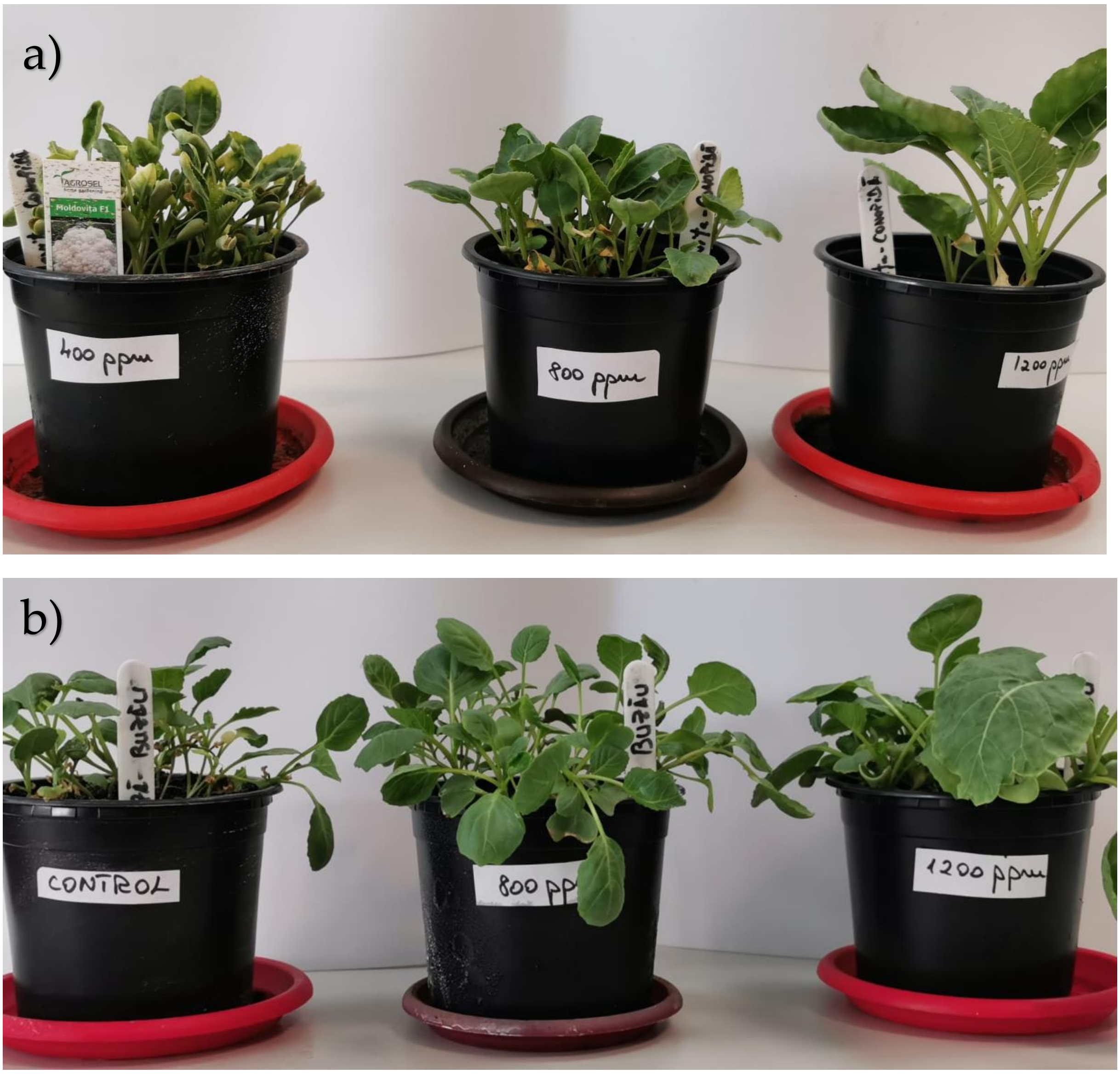

Figure S1. Plants from Green Cabbage (*Brassica oleracea* var. *capitata*, Varza de Buzau (Sem-Luca, Timisoara, Romania) (a) and Cauliflower (*Brassica oleracea* var. *botrytis*, Moldovita F1 (Agrosel, Campia-Turzii, Romania) (b) grown at 400 (control), 800 and 1200 ppmv carbon dioxide.
